# A possible case of renal oxalate deposit reported in an African fruit bat (*Epomops franqueti*)

**DOI:** 10.1080/23144599.2020.1807816

**Published:** 2020-08-19

**Authors:** O. O. Aina, M. A. Olude, F. E. Olopade, A. Balkema-Buschmann, M. H. Groschup, R. Ulrich, J. O. Olopade

**Affiliations:** a Department of Veterinary Anatomy, Faculty of Veterinary Medicine, University of Ibadan, Ibadan, Nigeria; b Department of Anatomy, Faculty of Basic Medical Sciences, University of Ibadan, Ibadan, Nigeria; c Institute of Novel and Emerging Infectious Disease, Friedrich Loeffler Institute, Isle of Riems, Germany; d Department of Experimental Animal Facilities and Biorisk Management, Friedrich Loeffler Institute, Isle of Riems, Germany; e Institute of Veterinary-Pathology, Faculty of Veterinary Medicine, Leipzig University, Leipzig, Germany

**Keywords:** *Epomops franqueti*, oxalate nephrosis, renal disease

## Abstract

We report a possible spontaneous case of oxalate nephrosis in an African fruit bat (*Epomops franqueti*), incidentally observed in Ibadan, South-West Nigeria, in an anatomical and serological survey of the species. Wild caught bats underwent sedation, intracardial perfusion, necropsy and histopathology. All 15 wild-caught African fruit bats were apparently healthy. However, light microscopy revealed mild oligofocal tubulonephrosis with intraluminal deposition of polarizing crystals interpreted as subclinical oxalate nephrosis in one case. In summary, we suggest a dietary aetiology, based on seasonal availability of high ascorbic acid or oxalate containing fruits. However, exposure to anthropogenic contaminants cannot be completely ruled out.

## 1. Introduction

Renal dysfunctions have hardly been documented in fruit bats. Renal toxicosis has been reported after topical administration of Ivermectin in dog-faced fruit bats, *Cynopterus brachyotis* [1]. Another case of renal involvement was in an experimental hendra virus infection in pregnant guinea-pigs and fruit bats (*Pteropus poliocephalus*) [2]. Oxalate nephrosis is a renal dysfunction characterized by the deposition of calcium oxalate crystals in the renal tubules with pale renal corticomedullary streaks grossly. This condition arises from the imbalance between intratubular calcium oxalate deposition and the body’s excretory capacity [3,4]. They may be a result of genetic defects in glyoxylate metabolism causing increased hepatic endogenous production of oxalate [3,5]. Endogenous deposition of oxalate also occurs following the normal degradation of glycine, an important constituent amino acid of collagen, elastin, hydroxyproline and serine [6] or increased absorption due to low intestinal calcium content [7]. Oxalate can also be produced endogenously as a metabolic by-product of ascorbic acid catabolism [3,4,6].

Various instances of primary oxalate nephrosis have been reported in humans [8–10]. Moreover, accidental ethylene glycol toxicity in humans, experimental exposure of domestic animals [11–13], as well as chronic renal disease [14,15] may cause these pathologic changes. A high percentage of Koala populations in parts of Australia have also been reported to come down with idiopathic oxalate nephrosis [16] and recently, oxalate-related renal disease was suggested as a potential cause of acute renal failure in a young cheetah [17].

This paper presents an incidental histological finding of a possible spontaneous renal oxalate deposition in an African fruit bat (*Epomops franqueti*), one of the samples caught in South-West Nigeria, in an anatomical and serological survey of the species.

## 2. Material and methods

A total of 15 African fruit bats (*Epomops franqueti*) were caught in University of Ibadan for an ongoing histological and serological study. Ethical approval was obtained by University of Ibadan – Animal Care and Use Research Ethics Committee (UI-ACUREC/App/2016/015). Bats were anaesthesized with ketamine (90 mg/kg) and xylazine (10 mg/kg) based on the work of Wirawati et al. [18] and then intracardially perfused with 10% neutral buffered formalin (NBF). Organ samples (including kidney, heart, brain, liver, spleen, pancreas, gastrointestinal tract, lungs blood vessels and lymph nodes) were fixed in 10% NBF and then embedded in paraffin, sectioned at 5 µm, and stained with Haematoxylin and Eosin based on routine method of Roulet [19] and Romeis [20] for light microscopy. Polarized light was used for confirmation of the oxalate crystals using a Zeiss Axio Scope.A1 and differential interference contrast illumination (Carl Zeiss Microscopy GmbH, Jena, Germany).

## 3. Histopathological findings

**Figure 1.**
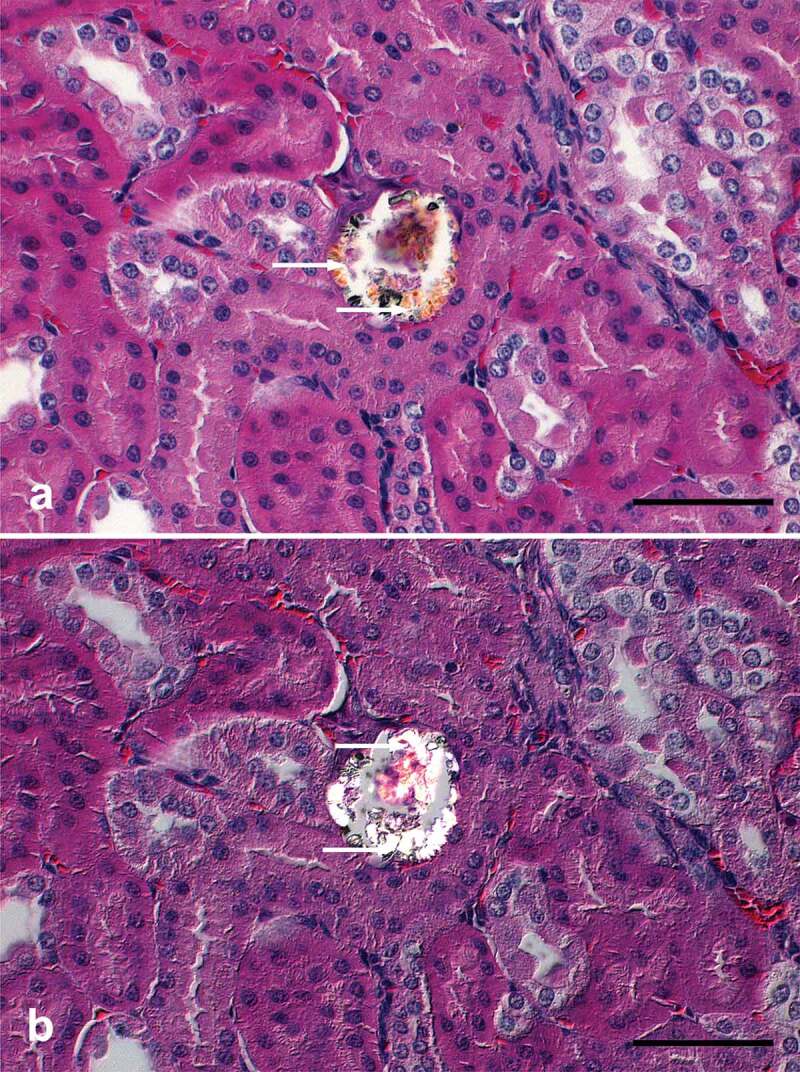
(a) Bright field light microscopy of H&E section of the kidney of male adult *Epomops franqueti* showing intraluminal yellow/orange, translucent, variably shaped crystals (arrows) (Bar: 50 µm). (b) Polarized light microscopy of the same section showing rhomboid and highly refractive crystals (arrows), without a definite arrangement in the clusters (Bar: 50 µm)

Only one of the 15 observed African fruit bats displayed mild, oligofocal tubular degeneration with intraluminal yellow/orange, translucent, fan-shaped to irregular crystals (Figure 1(a)). Furthermore, polarized light microscopy revealed birefringence of the intraluminal crystals (Figure 1(b))

## 4. Discussion

This to the best of our knowledge is the first report of renal tubular degeneration with luminal birefringent crystal deposit suggestive of oxalate nephrosis in fruit bats in the tropics. There is a strong possibility that the condition has dietary origin, based on seasonal availability of fruits with high oxalate content or its substrate, ascorbic acid. Oxalate nephrosis is a *sequelae* of precipitation of calcium oxalate crystals in the renal tubules. Ascorbic acid catabolism is one of the endogenous sources of oxalate deposition [3, 4, and 7]. Ascorbic acid intake through dietary sources of fruits and vegetables by wild primates and frugivorous bats in a tropical area was shown to be much greater than that of most human populations [21]. Consumption of plant-based diets and products containing high amounts of ascorbic acid have been discussed as the cause of oxalate nephrosis of herbivores and some other omnivores [22–25]. In an ecological study, of the same site studied, the wild fruits of heart-leaved fig (*Ficus polita*) and sponge gourd (*Luffa cylindrica*) were retrieved from captured fruit bats [26]. Moreover, sites of citrus species and watermelon (*Citrullus lanatus*) cultivation also exist within the vicinity. However, exposure to anthropogenic contaminants such as glycol, which is commonly used as antifreeze in the cooling systems of combustion engines, cannot be ruled out.

## 5. Conclusion

We observed a mild, oligofocal tubulonephrosis with crystal deposition in an African fruit bat, suggestive of a mild oxalate nephrosis. Further studies are needed to get more knowledge in this regard.
